# Utilizing network pharmacology to explore potential mechanisms of YiSui NongJian formula in treating myelodysplastic syndrome

**DOI:** 10.1080/21655979.2021.1933867

**Published:** 2021-06-08

**Authors:** Lerong Qin, Haiyan Chen, Xiaoqing Ding, Ming Guo, Haiyan Lang, Junxia Liu, Ling Li, Jing Liao, Junyao Liao

**Affiliations:** a Beijing University of Chinese Medicine, Beijing, China; b Department of Hematology, Dongfang Hospital Affiliated to Beijing University of Chinese Medicine, Beijing, China

**Keywords:** YiSui nongjian formula, network pharmacology, myelodysplastic syndrome

## Abstract

The study aims to explore potential mechanisms of YiSui NongJian formula (YSNJF) in treating myelodysplastic syndromes (MDS) by network pharmacology-based strategy. Active compounds and corresponding potential therapeutic targets of YSNJF were harvested by utilizing the database of TCMSP (Traditional Chinese Medicine Systems Pharmacology) and BATMAN-TCM (Bioinformatics Analysis Tool for Molecular mechanism of Traditional Chinese Medicine). MDS targets were adopted from GeneCard, KEGG (Kyoto Encyclopedia of Genes and Genomes), TTD (Therapeutic Target Database), DrugBank, and DisGeNet. Then a network of YSNJF- compounds-target-MDS network was harvested. The protein–protein interaction (PPI) network was then generated by the Sting database and subjected to Cytoscape software to harvest major and core targets network by topological analysis. Genes from the core targets network were further subjected to Gene Ontology (GO) and KEGG enrichment analysis to figure out potential targeting pathways. Finally, a compounds-targets-pathways network was generated by Cytoscape. A total of 210 active compounds and 768 corresponding potential therapeutic targets were harvested from ingredients of YSNJF. MDS was shown to have 772 potential treating targets with 98 intersected targets corresponding to 98 active compounds in YSNJF. Topological analysis revealed that 15 targets formed the core PPI network. Further, GO and KEGG enrichment analysis revealed that those core targets were mainly enriched on cell cycle- and immune-related pathways. The present study revealed that therapeutic effects of YSNJF on MDS might be achieved through regulating cell cycle- and immune-related pathways.

## Graphic abstract

**Figure:**
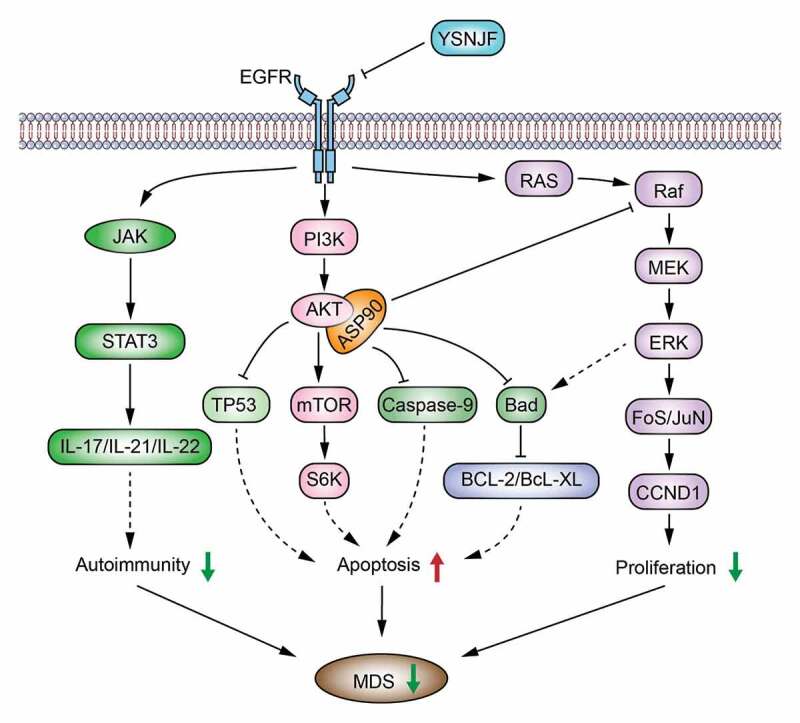

## Introduction

Myelodysplastic syndromes (MDS) are a group of clonal bone marrow neoplasms characterized by clonal proliferation of hematopoietic stem cells, ineffective hematopoiesis, myelodysplasia, peripheral blood cytopenia, recurrent genetic abnormalities, and a high risk of evolution to acute myeloid leukemia (AML), and manifested by morphologic dysplasia in hematopoietic cells and by peripheral cytopenia [1–4]. For patients diagnosed with MDS, the most effective treating method was allogeneic hematopoietic stem-cell transplantation (allo-HSCT) if they were eligible [5]. If patients were ineligible for allo-HSCT, active surveillance, erythropoiesis-stimulating agents, immunosuppressive therapy, red-cell transfusion, and iron chelation were given to low-risk MDS patients; hypomethylating agents, intensive chemotherapy, targeted therapies, and supportive care were given to higher-risk MDS patients according to their conditions [2]. Those treatments certainly have their limitations, for example, allo-HSCT was often seen with scarce donor, limited indications to apply, and were reported to be associated with substantial morbidity and mortality; chemotherapy utilized were shown to induce large economic burden to patients with drug resistance and therapy-related complications frequently happens.

Traditional Chinese herbal medicine has been widely adopted for various disease treatments for more than 2,000 years. Currently, integrative medicine by combining traditional Chinese medicine with western medicine was shown promising therapeutic effects with properties of economical and practical [6]. YiSui NongJian formula (YSNJF), authorized by prof. Su Wei (Department of Hematology, Dongfang Hospital Beijing University of Chinese Medicine) and generated in the 1980s, is composed of 20 herbal medicines, including zhihuangqi (*Radix Astragali preparata*), dangshen (*Codonopsis pilosula*), baizhu (*Atractylodes macrocephala Koidz*.), fuling (*Poria cocos*), shuizhi (*Hirudo*), etc. From our previous clinical practice, we have found that YSNJF together with chemotherapy could significantly improve hemogram parameter and MDS symptoms, which could further promote the prognosis of MDS patients when compared with chemotherapy alone, especially in low-risk MDS patients [7–9]. Although the effective rate of YSNJF treatment is not significantly higher than that of granulocyte-colony stimulating factor (G-CSF) treatment (70.00% vs 59.1%, 23 MDS patients in each group, P > 0.05), YSNJF treated patients had a much lower serum concentration of sICAM-1 and HIF1α than those of G-CSF treatment [8]. However, the underlying mechanism of therapeutic effects of YSNJF in MDS treatment remains largely unknown.

Network pharmacology for exploring mechanisms of traditional Chinese herbal medicine, as first proposed by Li et al. [10], was able to construct a drug-target network and explore potential mechanisms of drug action based on structural similarities of active compound and target proteins [11]. Applications of this systematic biology could help to reveal pharmacological action, mechanism of action, and safety of TCMs and currently is a hotspot for explaining treatment activities of TCM [12].

Therefore, the present study was designed to determine the potential mechanisms of ingredients in YSNJF in treating MDS by utilizing the method of network pharmacology. Our research may help in MDS drug screening, improve the formulation of YSNJF, and may provide a useful reference for the exploration the therapeutic mechanism of other traditional Chinese medicine.

## Methods

### Screening of active compounds and corresponding potential therapeutic targets of YSNJF

Two online databases, TCMSP [13] (Traditional Chinese Medicine Systems Pharmacology, https://tcmspw.com/tcmsp.php) and BATMAN-TCM [14] (Bioinformatics Analysis Tool for Molecular mechanism of Traditional Chinese Medicine, http://bionet.ncpsb.org/batman-tcm/), were utilized for screening of active compounds and corresponding potential therapeutic targets of YSNJF. Herbal medicines in YSNJF were firstly subject to TCMSP, oral relative bioavailability (OB) ≥ 30%, and drug-likeness (DL) ≥ 0.18 were set as threshold for active compounds and corresponding potential therapeutic targets screening. And if herbal medicines in YSNJF were not included in TCMSP, those herbal medicines were then subject to BATMAN-TCM. The screening threshold in BATMAN-TCM was set as drug-target similarities score ≥ 20 and adjusted *P*-value < 0.05.

### MDS targets screening

MDS targets screening were performed by search several databases like GeneCard (https://www.genecards.org/), KEGG (Kyoto Encyclopedia of Genes and Genomes, https://www.kegg.jp/), TTD [15] (Therapeutic Target Database, http://bidd.nus.edu.sg/group/cjttd/), DrugBank (https://go.drugbank.com/), and DisGeNet (https://www.disgenet.org/) with the term of ‘Myelodysplastic Syndrome/myelodysplastic syndrome’. Duplicated targets were removed to harvest the final MDS target sets.

### Construction of YSNJF-compounds-target-MDS network

The harvested YSNJF-related targets and MDS-related targets were subjected to a Venn diagram to find out intersected targets so that corresponding active compounds could also be recognized. Then the YSNJF-compounds-target-MDS network was constructed by Cytoscape 3.7.2. Nodes in the network were a representation of YSNJF, MDS, and harvested compounds and targets. Edges in the network represent the relationship of each node and the quantities of each edge were defined as ‘degree’.

***Protein–protein interaction (PPI) network construction and major and core targets network identification***

PPI network of intersected targets was generated by uploading intersected targets to the online database of String (https://string-db.org). Minimum required interaction score was set as ‘medium confidence = 0.4’ while other parameter remains as default. Then generated PPI network was loaded to Cytoscape. A build-in module of CytoCNA was further utilized to generate major targets network when Betweenness, Closeness, Degree/Connectivity, Eigenvector, Local Average Connectivity-based method, and Network of each node were all larger than the median. Core target networks were generated from major target networks by topological analysis.

### Gene ontology (GO) and KEGG enrichment analysis of intersected targets

To better interpret the function of those intersected targets, pathway enrichment analyzes by GO and KEGG were performed by using Metasacpe (http://metascape.org/). Cutoff values in this database were set as follows: Min Overlap = 3; Min Enrichment>1.5; *P*-Value Cutoff = 0.01. Top 20 enriched pathways of GO (including biological process, cellular component, and molecular function) and KEGG were made visualized in a bubble chart by utilizing an online bioinformatics tool (bioinformatics, http://www.bioinformatics.com.cn/). The size of the bubble represents the number of targets enriched in the indicated pathway and the color of the bubble represents the *P*-value of enrichment.

### Construction of compounds-targets-pathways network

Top 20 enriched KEGG pathways from enrichment analysis, together with corresponding compounds and target were then loaded into Cytoscape software. By separately adjust the plot, a visualized compounds-targets-pathways network was generated.

## Results

### Screening of active compounds and corresponding potential therapeutic targets of YSNJF

As presented in Table 1, active compounds and corresponding potential therapeutic targets if herbal medicines from YSNJF were harvested from TCMSP (top 15 herbal medicines presented in Table 1) and BATMAN-TCM (rest 5 herbal medicines) database. The harvested targets were then verified by Uniprot (https://www.uniprot.org/) to exclude invalid compound-target pairs. Finally, a total of 210 active compounds and 768 corresponding targets were generated. Some of the compounds were found to have to exist in many herbal medicines and to have multiple targets (Table 2).

**Table 1.** Active compounds and corresponding potential therapeutic targets of YSNJF

Herbal medicines	Validated compounds (N)	Invalidated compounds (N)	Intersected compounds (N)	Targets (N)
Huangqi (HQ)	19	3	6	390
Dangshen (DS)	21	4	2	185
Chengpi (CP)	5	0	1	76
Baizhu (BZ)	7	3	1	20
Dihuang (DH)	2	0	2	32
Danggui (DG)	2	0	2	63
Baishao (BS)	13	5	4	107
Fuling (FL)	15	9	0	26
Chuangqiong (CQ)	7	2	3	31
Heshouwu (HSW)	25	9	0	292
Danshen (DanS)	65	7	1	795
Tusizi (TSZ)	11	1	5	299
Gouqizi (GQZ)	45	10	7	329
Roucongrong (RCR)	7	0	2	194
Taoren (TR)	23	4	2	102
Honghua (HH)	22	6	6	395
Lujiao (LJ)	2	0	0	13
Guijia (GZ)	1	0	0	1
Tubiechong (TBC)	1	0	0	32
Shuizhi (SZ)	15	2	0	426
Total	308	65	44	3808

Notes: Huangqi (HQ): *Astragalus*; Dangshen (DS): *Codonopsis pilosula*; Chengpi (CP): *Pericarpium Citri Reticulatae*; Baizhu (BZ): *Atractylodes macrocephala koidz*.; Dihuang (DH): *Rehmannia glutinosa*; Danggui (DG): *Angelica sinensis*; Baishao (BS): *radix paeoniae alba*; Fuling (FL): *Poria cocos*; Chuangqiong (CQ): *Chuanxiong Rhizoma*; Heshouwu (HSW): *polygonum multiflorum thumb*; Danshen (DanS): *Salvia miltiorrhiza*; Tusizi (TSZ): *semen cuscutae*; Gouqizi(GQZ): *fructus lycii*; Roucongrong (RCR): *Cistanche deserticola*; Taoren (TR): *semen persicae*; Honghua (HH): *safflower*; Lujiao (LJ): *Cornu Cervi*; Guijia (GJ): *tortoise shell*; Tubiechong (TBC): *eupolyphaga*; Shuizhi (SZ): *Hirudo*.

**Table 2.** Intersected compound from multiple herbal medicines

Label of compounds	Name of compounds	Number of targets (N)	Source of herbal medicines
M1	Kaempferol	55	HQ, GQZ, BZ, TSZ, HH
M2	Quercetin	140	HQ, GQZ, TSZ, HH, RCR
M3	Stigmasterol	29	DS, GQZ, DH, DG, HH
M4	Luteolin	52	DS, DanS, HH
M5	Beta-sitosterol	34	BS, DG, TSZ, GQZ, TR, HH, RCR
M6	Mairin	1	HQ, BS
M7	(3S,8S,9S,10 R,13 R,14S,17 R)-10,13-dimethyl-17-[(2 R,5S)-5-propan-2-yloctan-2-yl]-2,3,4,7,8,9,11,12,14,15,16,17-dodecahydro-1 H-cyclopenta[a]phenanthren-3-ol	1	HQ, BS
M8	Sitosterol	3	CP, DH, CQ, BS
M9	Folic acid	1	HQ, CQ
M10	Isorhamnetin	28	HQ, TSZ
M11	Cholesterol	3	GQZ, TSZ, HH
M12	Mandenol	3	GQZ, TR
M13	Sitosterol alpha1	5	GQZ, TR

### MDS targets screening

**Figure 1.**
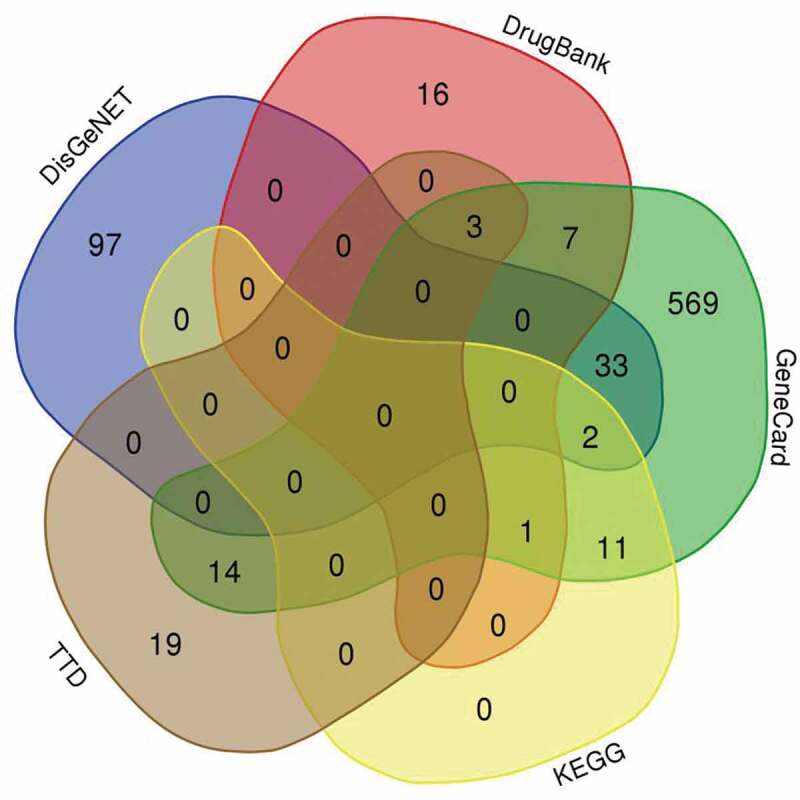
Harvested 772 MDS-related targets from indicated databases

Subjecting search term ‘Myelodysplastic Syndrome/myelodysplastic syndrome’ to GeneCard, TTD, DrugBank, DisGeNET, and KEGG have found 640, 40, 36, 948, and 15 targets, respectively. After verification by Uniprot and movement of duplicated targets, a total of 772 MDS-related targets were finally obtained (Figure 1).

### Construction of YSNJF-compounds-target-MDS network

**Figure 2.**
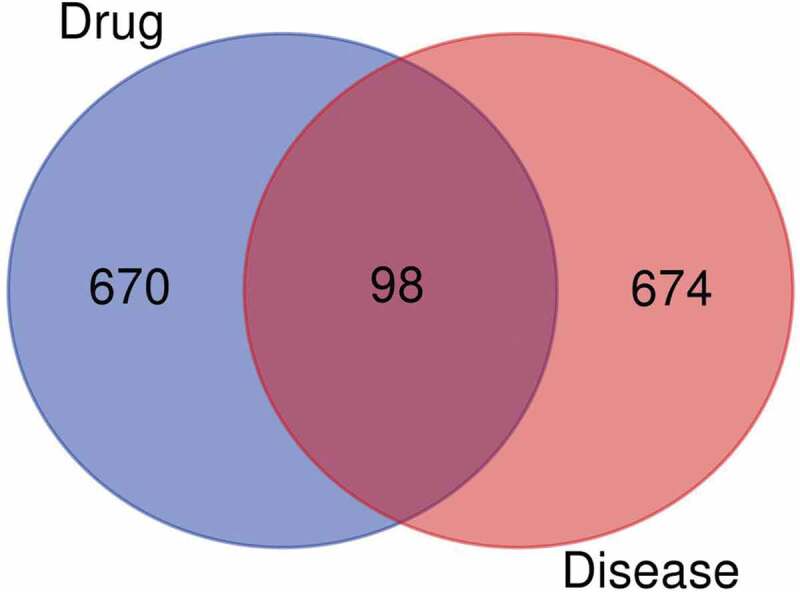
Venn plots show intersected targets between drug and disease

**Figure 3.**
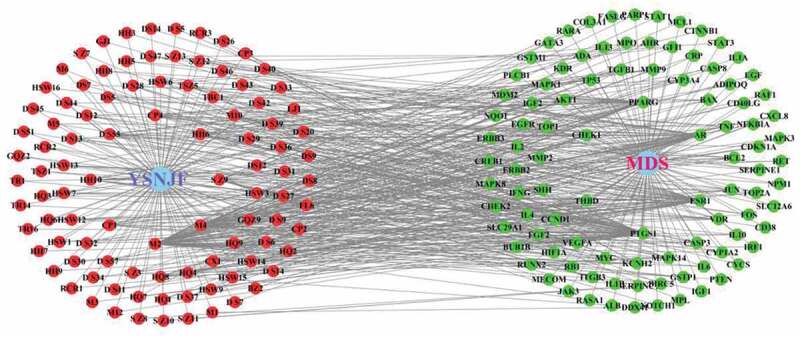
YSNJF-compounds-target-MDS network. Blue nodes represent drug and disease, respectively; red nodes represent active compounds; green nodes represent disease-related targets. Lines in the figure represent the interaction between two nodes

After the YSNJF-related and MDS-related targets were obtained, the intersected targets were determined by the Venn plot (Figure 2). Compounds that peculiar to specific herbal medicines were labeled as herbal names with numbers while compounds commonly existed in several herbal were labeled as previously presented in Table 2. A YSNJF-compounds-target-MDS network was constructed by Cytoscape (Figure 3). In the network, 98 active compounds with 98 corresponding intersected targets were found. More importantly, by further analyze the degrees of each node, we have found that quercetin, luteolin, crocetin, baicalein, nobiletin, and tanshinone IIb could potentially be the most important active compounds of YSNJF. Core targets as determined in the same manner have also revealed that prostaglandin-endoperoxide synthase 1 (PTGS1), estrogen receptor 1 (ESR1), androgen receptor (AR), peroxisome proliferator-activated receptor γ (PPARG), and thrombomodulin (THBD) were potentially key targets for MDS treatment (Figure 3 and Table 3).

**Table 3.** List of major compounds corresponding to intersected targets

Label of compounds	Name of compounds	Number of targets	Label of compounds
M2	Quercetin	58	AHR, AKT1, AR, BAX, BCL2, BIRC5, CASP3, CASP8, CCND1, CD40LG, CDKN1A, CHEK2, COL3A1, CRP, CXCL8, CYP1A2, CYP3A4, EGF, EGFR, ERBB2, ERBB3, FOS, GSTM1, GSTP1, HIF1A, IFNG, IGF2, IL10, IL1A, IL1B, IL2, IL6, IRF1, JUN, KCNH2, MAPK1, MMP2, MMP9, MPO, MYC, NFKBIA, NQO1, PARP1, PPARG, PTEN, PTGS1, RAF1, RASA1, RB1, RUNX2, SERPINE1, STAT1, TGFB1, THBD, TNF, TOP1, TOP2A, TP53
M4	Luteolin	29	MMP2, BIRC5, GSTP1, TNF, CCND1, VEGFA, MCL1, IL6, CASP3, IL10, MAPK1, MDM2, EGFR, IL2, ERBB2, IFNG, IL4, TOP2A, RB1, TP53, CDKN1A, AKT1, NFKBIA, JUN, AR, CD40LG, MMP9, TOP1PPARG
S Z9	Crocetin	18	VDR, FASLG, RARA, GATA3, PLCB1, MECOM, JAK3, ITGB3, IL1B, PTGS1, AR, NOTCH1, ADIPOQ, PPARG, IGF1, RET, IL13, IGF1
HH6	Baicalein	15	CYCS, BCL2, AHR, MPO, VEGFA, CASP3, IGF2, FOS, HIF1A, TP53, AKT1, PTGS1, AR, BAX, MMP9
DanS55	(6S)-6-hydroxy-1-methyl-6-methylol-8,9-dihydro-7 H-naphtho[8,7-g]benzofuran-10,11-quinone	13	BCL2, NPM1, MYC, CASP3, CYP1A2, FOS, TP53, CDKN1A, ITGB3, NFKBIA, JUN, CYP3A4, MMP9
CP4	Nobiletin	13	CHEK1, BCL2, KCNH2, ESR1, CREB1, MAPK8, TP53, JUN, AR, BAX, MMP9, PPARG, PTGS1
HH10	Beta-carotene	10	MMP2, BCL2, CTNNB1, VEGFA, MYC, CASP3, CYP1A2, AKT1, JUN, CYP3A4
HQ8	Formononetin	9	CHEK1, THBD, ESR1, IL4, JUN, AR, PPARG, PTGS1, MAPK14
CP1	Naringenin	9	BCL2, GSTP1, ESR1, CASP3, AKT1, ADIPOQ, PPARG, PTGS1, MAPK3
HQ4	7-O-methylisomucronulatol	8	CHEK1, KCNH2, THBD, ESR1, AR, PTGS1, PPARG, MAPK14
CX1	Myricanone	8	CHEK1, CHEK1, KCNH2, THBD, ESR1, KDR, AR, PPARG, MAPK14

### PPI network construction and major and core targets network identification

**Figure 4.**
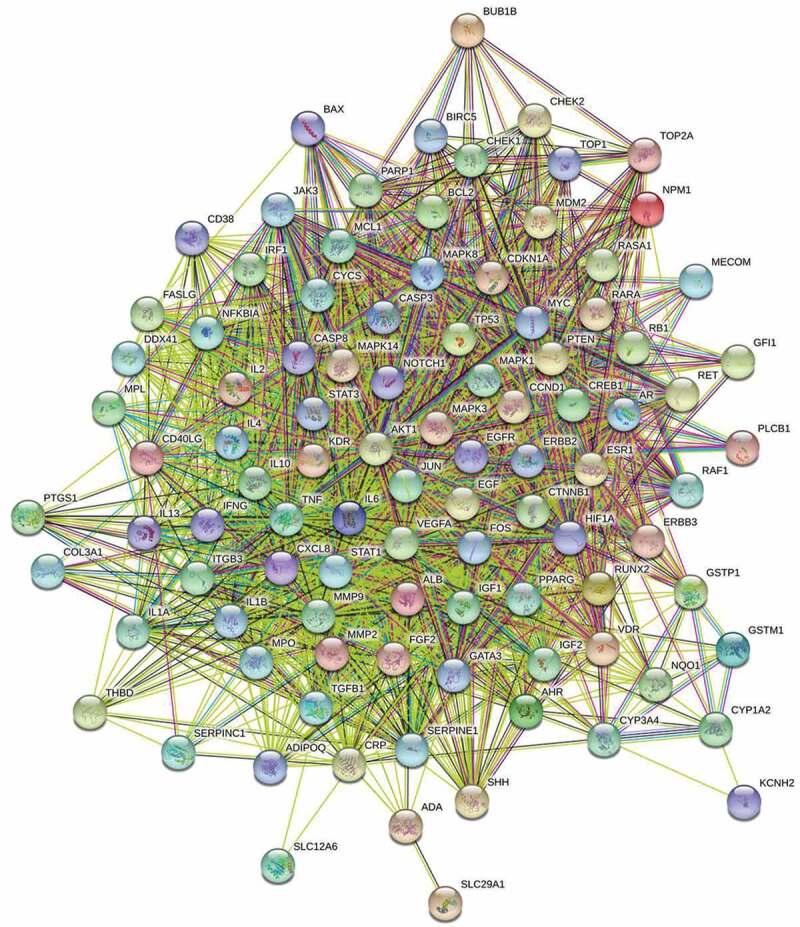
Protein–protein interaction (PPI) network of drug and disease intersected targets

**Figure 5.**
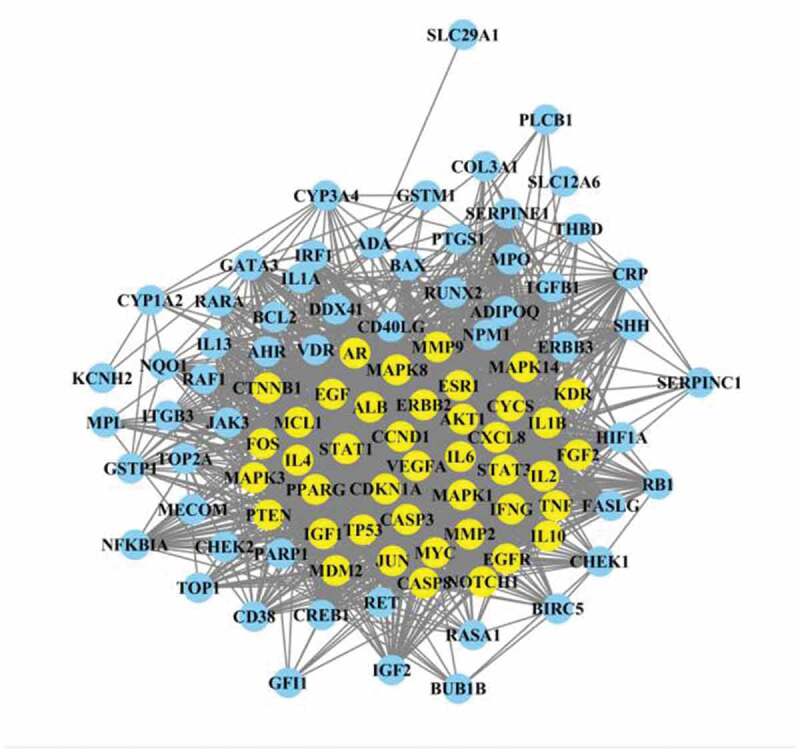
Protein–protein interaction (PPI) network of identified major targets. Light blue nodes were regular targets while yellow nodes were major targets

**Figure 6.**
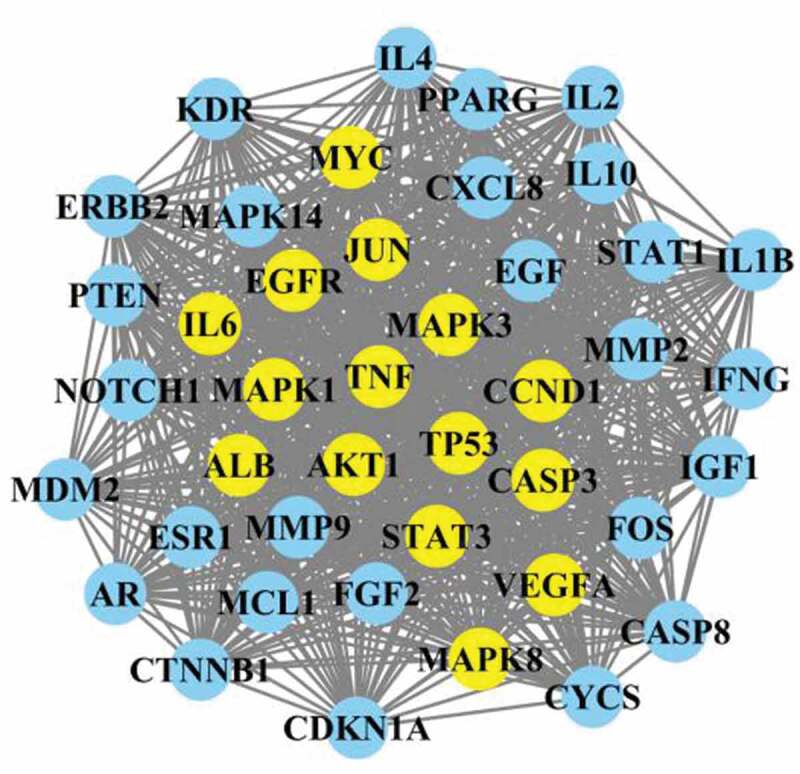
Protein–protein interaction (PPI) network of identified core targets. Light blue nodes were regular targets while yellow nodes were core targets

The intersected targets were further subjected to PPI network construction (Figure 4). Those networks were constructed based on evidence from text mining, experiments, databases, co-expression, neighborhood, gene Fusion, and co‑occurrence as indicated on the String database. As determined by String, the average node degree of constructed network was 40.3, and the top targets with the highest node degrees were mitogen-activated protein kinase 1(MAPK1), G1/S-Specific Cyclin-D1 (CCND1), tumor protein P53(TP53), MAPK3, signal transducer and activator of transcription 3 (STAT3), interleukin 2 (IL-2), THBD and epidermal growth factor receptor (EGFR). String harvested PPI were then further analyzed by Cytoscape. The major targets network (Figure 5) were generated with cutoff values set as following: node = 42, edge = 799, degree median = 62. And core targets network was generated by topological analysis (Figure 6). The topological analysis obtained targets were following String-determined top targets.

### GO and KEGG enrichment analysis of intersected targets

**Figure 7.**
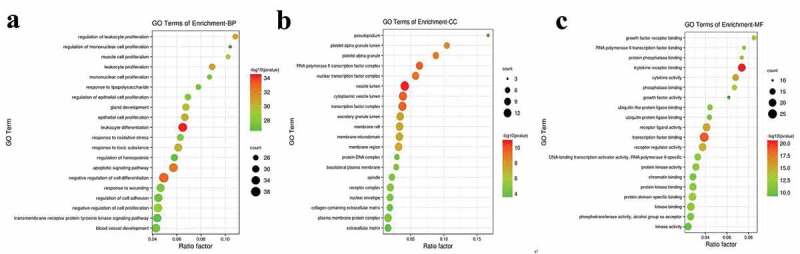
GO enrichment analysis of intersected targets between drug and disease. The top 20 GO terms in biological process (BP, A), cellular component (CC, B), molecular function (MF, C) with adjusted *P* value <0.05 were selected and present in a bubble chart manner. The size of bubble represents number of targets enriched in the indicated pathway and the color of the bubble represents the *P* value of enrichment

**Figure 8.**
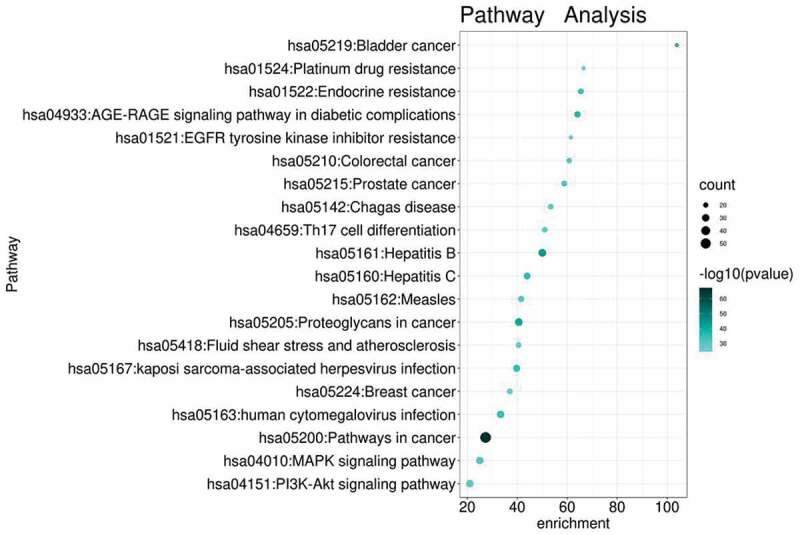
KEGG enrichment analysis of intersected targets between drug and disease. The top 20 KEGG pathways with adjusted *P* value <0.05 were selected and present in a bubble chart manner. The size of bubble represents the number of targets enriched in the indicated pathway and the color of the bubble represents the *P* value of enrichment

**Figure 9.**
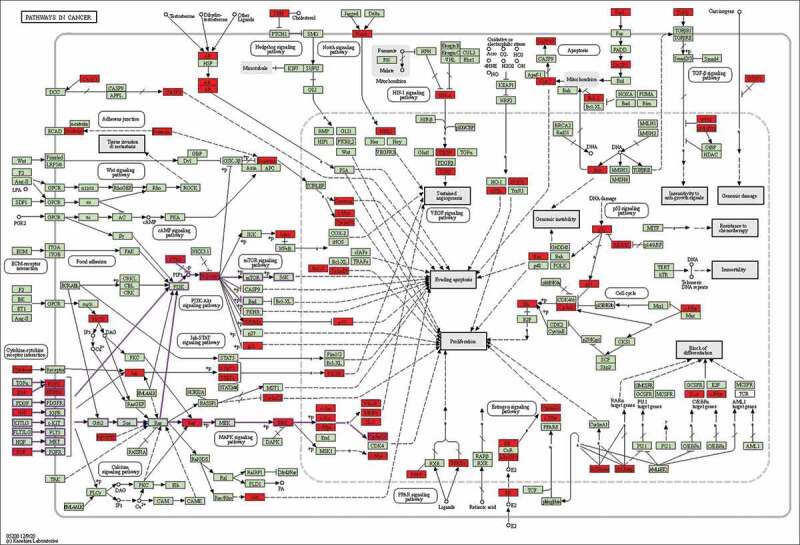
Representative signaling transduction of Pathways in cancer merged with identified targets. Genes in red are potential targets of YSNJF in treating MDS as predicted by network pharmacology

**Figure 10.**
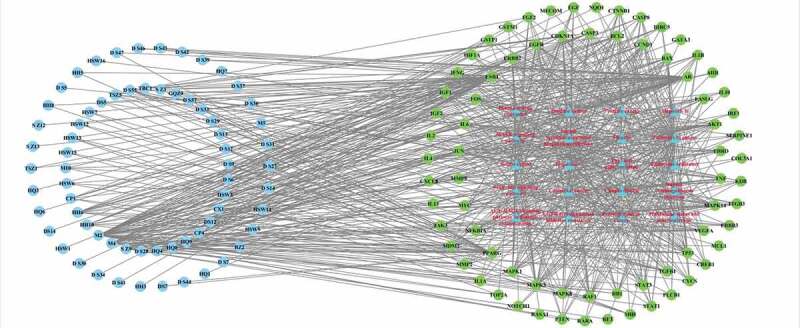
Compounds-targets-pathways network. Light blue nodes represent active compounds; red triangle nodes represent KEGG pathways; green nodes represent disease-related targets. Lines in the figure represent the interaction between two nodes

Targets from the core network were then undergone enrichment analysis by GO and KEGG. Top enriched GO terms (Figure 7) were majorly focused on cell differentiation-related and immune-related pathways, like leukocytes differentiation, negative regulation of cell differentiation, apoptotic signaling pathway, etc. KEGG enrichment analysis (Figure 8) revealed that cancer-related and immune-related pathways together with specific signal transduction pathways were among the top enriched pathways, like pathways in cancer (Figure 9), hepatitis B/C, MAPK (mitogen-activated protein kinase) signaling pathway, PI3K (phosphoinositide 3-kinase)-AKT signaling pathway, and Th17 cell differentiation, etc. Interestingly, the signaling transduction of pathways in cancer was mainly composed of genes from MAPK and PI3K-AKT signaling pathways, further suggesting the importance of those pathways in the pathogenesis of MDS (Figure 9). This result also reflects potential signaling pathway cross-regulation in treating MDS (Figure 9). Those results have revealed that the therapeutic effect of YSNJF on MDS may partly be achieved by its immune-modulation effects. Together with the above-mentioned results, a compounds-targets-pathways network was further constructed. As presented in Figure 10, multiple active compounds from YSNJF could target multiple proteins and subsequently initiates complex signal transduction, like regulation of immune differentiation, negatively regulate cancer-related pathways, and then restrains the progression of MDS and promote the prognosis of patients.

## Discussion

In the current study, network pharmacology methods were used to explore potential targets and mechanisms of YSNJF in the treatment of MDS. A total of 210 active compounds were identified by using TCMSP (OB ≥ 30% and DL ≥ 0.18) and BATMAN-TCM (similarities score ≥ 20 and adjusted *P*-value < 0.05) and 768 corresponding targets were identified by using GeneCard, KEGG, TTD, DrugBank, and DisGeNet. 98 drug-related genes, which may be the targets of YSNJF in treating MDS, were harvested by using a Venn tool. PPI network of compounds-targets was then generated and core targets were identified by using Cytoscape together with String database. GO and KEGG enrichment analysis of 15 core targets revealed that PI3K/AKT-, MAPK/ERK-, and JAK/STAT3-mediated cell cycle- and immune related-signaling pathway were the most enriched ones. Therefore, genes in those signal pathways may be critical targets for MDS treatment.

Traditional Chinese Medicine (TCM) has been widely adopted in China and surrounding countries for thousands of years [16]. Of note, TCM had made a great contribution in the fight against the COVID-19 pandemic in prevention and mild infection control [17]. Diseases treated by TCM were mainly achieved by a good many active compounds that worked synergistically. This working principle also promotes the difficulties in identifying the specific molecular mechanism of therapeutic effects achieved by TCM. However, with the development of muti-omics and bioinformatic analysis methods, network pharmacology was invented. Network pharmacology was known to construct a drug-target network so that the underlying mechanisms of TCM could be revealed. YSNJF, a formula with 20 herbal medicines, was originated from bazhen decoction and guilu erxian decoction, two famous TCM formulas for hematologic disease treatment. Although YSNJF were shown promising therapeutic effects for MDS treatment in the clinic, the mechanism has never been explored before. The present study has partly revealed the mechanism of YSNJF in the treatment of MDS by using network pharmacology, which might provide a reference for future research of specific mechanism exploration of YSNJF and other TCM formulas.

As previously reported that natural products derived products were able to fight against cancer-like polysaccharides, proteins, and organelles from fungus could be utilized for anti-cancer drug screening [18], herbal derived active compounds were also reported to inhibit tumor growth. In our current study, the top-scored active compound obtained from intersected targets was consistent with results from pathway analysis. Generally, from our current research, quercetin, luteolin, crocetin, baicalein, nobiletin, and tanshinone IIb were the major active compound in the treatment of MDS. As previously reported, quercetin and crocetin were able to inhibit MAPK- or PI3K/AKT-mediated BCL-2 expression; promote the expression of caspase-3, caspase-8, caspase-9, and PARP so that tumor cells-cycle was inhibited, and apoptosis of tumor cell initiated [19]. Also, quercetin was reported to have the ability to modulate the Th17/Treg balance by inhibiting inflammasome activation as well as activating HO-1-mediated anti-inflammatory response [20]. Luteolin was also reported to have the ability to restrain cell cycle of tumor cells and inhibiting tumor cell proliferation via regulating MAPK- and PI3K/AKT-mediated signal transduction [21]. Additionally, drug resistance to tyrosine kinase inhibitor could be reversed by luteolin through reversing the epithelial-mesenchymal transformation process [22]. Similar anti-tumor effects by inhibiting tumor cell proliferation, promoting tumor cell apoptosis, and restraining tumor metastasis could also be achieved by crocetin [23], baicalein [24,25], tanshinone IIb [26,27], and nobiletin [28,29]. Together with our findings, researchers suggested that therapeutic effects of YSNJF to MDS may partly through regulating tumor proliferation-related pathways.

Topological analysis harvested core targets network also revealed some clues which may help to understand the therapeutic action of YSNJF. TP53, a typical tumor-inhibiting gene, was reported to upregulating the expression of BAX while downregulating Bcl-2 so that it could promote target cell apoptosis. AKT serine/threonine kinase 1 (AKT1), also known as protein kinase B alpha (PKB alpha), was demonstrated to be involved in process of cell survival, proliferation, and anti-apoptosis once phosphate by PI3K signal pathway. MAPKs could be activated by various cytokines and extracellular stimulus and MAPKs signal pathways were shown to be critically involved in cell differentiation and proliferation. MAPK1 (ERK1) and MAPK3 (ERK2) were the most important MAPKs for signal transduction. CCND1, whose up-and-down-regulation could profoundly change the course of the cell cycle, is a vital gene in the cell cycle, especially in the process for cell from the G1 phase to the S phase. EGFR, a protein encoded by this gene is a tyrosine-protein kinase, was shown to participate in cell migration via regulating Ras-MAPK-ERK and PI3K-AKT signal pathways. Those results further suggested that MAPK and PI3K-AKT signal pathways were profoundly regulated by YSNJF in the treatment of MDS.

The above-mentioned analysis was further validated by enrichment analysis from GO and KEGG. Particularly, KEGG enrichment analysis revealed that MAPK and PI3K-AKT signaling pathways were among the top enriched pathways. Research from patients of low-risk MDS has found that bone marrow stromal cells and MDS clonal cells secrets excessive hematopoietic inhibitory factors like TNF-α, IFN-γ, TGF-β, and VEGF. Those factors induce hyperphosphorylation of the p38 MAPK signaling pathway and subsequently induced the immoderate apoptosis of bone marrow hematopoietic stem/progenitor cells, especially the normal stem/progenitor cells [30,31]. As demonstrated by another research group, immoderate apoptosis of CD34^+^ cells was highly associated with over-activation of the p38 MAPK signaling pathway in low-risk MDS patients [32]. Another TCM formula (compound shenlu granules) was shown to inhibit hyperphosphorylation of the p38 MAPK signaling pathway, decrease immoderate apoptosis of CD34^+^ cells and improve ineffective hematopoiesis in low-risk MDS patients [32]. Mammalian target of rapamycin (mTOR) signaling, downstream of PI3K-AKT signaling activation, could be activated by phosphorylation of AKT to regulate autophagy process via mTOR-Atg1-Atg13 or mTOR-ULK1-Atg13 signal transduction under pressure stimulation or oxidative stress stimulation [33]. Aberrant autophagy process was reported to be closely related to the pathogenesis of cancer [34], including MDS, given the fact that autophagy plays critical roles in hematopoietic differentiation [33,35–38]. Clone formation assay of blood cells from low-risk MDS patients has revealed that excessive autophagy could be observed in clones of erythroid cells [39], suggested that abnormal erythropoiesis in MDS was highly related to aberrant autophagy. Further research also revealed that thrombocytopenia in low-risk MDS patients was caused by excessive autophagy induced programmed cell death in megakaryocytes at bone marrow [40]. Together with our findings, those researches suggested that the therapeutic effect of YSNJF in MDS treatment might be achieved by promoting tumor cell death and maintain homeostasis of the hematopoietic system through regulating MAPK and PI3K-AKT signaling pathways.

Another interesting finding from our result was that the therapeutic effects of YSNJF to MDS might also be achieved in an immunomodulatory manner. From our GO and KEGG analysis, we have found that leukocytes differentiation and Th17 cell differentiation signaling pathways were significantly enriched in the core targets network. Th17 cells were differentiated from CD4^+^ T cells. The transcriptional signature of Th17 cells was RORγt and these kind cells were known to secret pro-inflammatory cytokines, like IL-17, IL-21, and IL-22. Report has demonstrated that T cell-mediated immune dysfunction is a key characteristic of MDS [41]. Imbalanced Th17/Treg ratio characterized by upregulated Th17 cells and downregulated Treg cells could result in autoimmune abnormalities and further resulted in blood cell reduction [42,43]. Also, the number of Th17 cells was positively correlated with bone marrow cell deficiency. Inappropriately up-regulated PI3K-AKT signaling pathways could promote Th17 cell production by promoting HIF-1α expression, STAT3 phosphorylation, RORγt nuclear translocation, and down-regulating GFI1 expression [44].

Also, even not included in the current work, cancer early detection is a well-acknowledged critical pre-step for effective cancer treatment. Cost-effectiveness early detection method was of great need. An interesting work performed by Low et al. developed a smartphone-based electrochemical biosensing system for the detection of cancer biomarkers with good accuracy and recovery ratio [45], which is quite inspiring in cancer early detection.

This research has several limitations. Firstly, as proved by previous reports, signaling pathways could cross regulate each other, key signaling pathways involved in therapeutic effects of YSNJF to MDS still need to be explored. Secondly, works done in the current study were mainly obtained from the bioinformatic analysis, experimental validations both *in vitro* and *in vivo* could be adopted in future research. Finally, limited by current pharmacology technology, the dose-effect relationship between YSNJF and MDS was not able to be determined.

## Conclusion

By utilizing network pharmacology, the present study has revealed for the first time that therapeutic effects of YSNJF to MDS might be mainly achieved by quercetin, luteolin, crocetin, baicalein, nobiletin, and tanshinone IIb via regulating cell proliferation-related and immune-related signal pathways. Our research has provided a direction for specific mechanism exploration of YSNJF, several potential targets for MDS treatment, and a promising reference for exploring the mechanism of other TCM formulas.

## Data Availability

The data related to this research can be obtained from the corresponding author upon reasonable request.

## Highlights

(1) Quercetin, luteolin, crocetin, baicalein, nobiletin, and tanshinone IIb might be the major active compound in the treatment of MDS by YSNJF.

(2) Therapeutic effect of YSNJF against MDS might be achieved by altering cell cycle- and immune-related pathways.

(3) YSNJF might target JAK/STAT3-, MAPK/ERK-, and PI3K/AKT-mediated signaling pathway in treating MDS.
